# Evaluating coverage of maternal syphilis screening and treatment within antenatal care to guide service improvements for prevention of congenital syphilis in *Countdown* 2030 Countries

**DOI:** 10.7189/jogh.10.010504

**Published:** 2020-06

**Authors:** Shivika Trivedi, Melanie Taylor, Mary L Kamb, Doris Chou

**Affiliations:** 1 CDC Foundation, Atlanta, Georgia, USA; 2 World Health Organization, Geneva, Switzerland; 3 Centers for Disease Control and Prevention (CDC), Atlanta, Georgia, USA

## Abstract

**Background:**

Countdown to 2030 (CD2030) tracks progress in the 81 countries that account for more than 90% of under-five child deaths and 95% of maternal deaths in the world. In 2017, CD2030 identified syphilis screening and treatment during antenatal care (ANC) as priority indicators for monitoring.

**Methods:**

Country-reported data in the UNAIDS Global AIDS Monitoring System (GAM) system were used to evaluate four key syphilis indicators from CD2030 countries: (1) maternal syphilis screening and (2) treatment coverage during ANC, (3) syphilis seroprevalence among ANC attendees, and (4) national congenital syphilis (CS) case rates. A cascade analysis for CD2030 countries with coverage data for the number of women attending at least 4 antenatal care visits (ANC4), syphilis testing, seroprevalence and treatment was performed to estimate the number of CS cases that were attributable to missed opportunities for syphilis screening and treatment during antenatal care.

**Results:**

Of 81 countries, 52 (64%) reported one or more values for CS indicators into the GAM system during 2016-2017; only 53 (65%) had maternal syphilis testing coverage, 41 (51%) had screening positivity, and 40 (49%) had treatment coverage. CS case rates were reported by 13 (16%) countries. During 2016-2017, four countries reported syphilis screening and treatment coverage of ≥95% consistent with World Health Organization (WHO) targets. Sufficient data were available for 40 (49%) of countries to construct a cascade for data years 2016 and 2017. Syphilis screening and treatment service gaps within ANC4 resulted in an estimated total of 103 648 adverse birth outcomes with 41 858 of these occurring as stillbirths among women attending ANC4 (n = 31 914 408). Women not in ANC4 (n = 25 619 784) contributed an additional 67 348 estimated adverse birth outcomes with 27 198 of these occurring as stillbirths for a total of 69 056 preventable stillbirths attributable to syphilis in these 40 countries.

**Conclusion:**

These data and findings can serve as an initial baseline evaluation of antenatal syphilis surveillance and service coverage and can be used to guide improvement of delivery and monitoring of syphilis screening and treatment in ANC for these priority countries.

Syphilis is a leading cause of adverse pregnancy outcomes including stillbirth and neonatal death. In 2016, WHO estimated there were 988 000 maternal syphilis infections worldwide resulting in 355 000 adverse pregnancy outcomes, of which over half were stillbirths or neonatal deaths [1,2]. Both screening and treatment for syphilis during pregnancy remain sub-optimal in low- and middle-income countries despite diagnosis and prevention of maternal-to-child transmission (MTCT) of syphilis being feasible, inexpensive and cost-effective [3]. In 2007, WHO and partners launched a global initiative to eliminate congenital syphilis based on the pillars of 1) sustained political commitment and advocacy, 2) access to and quality of maternal and newborn health services; 3) universal syphilis screening for all pregnant women and treatment of women testing positive and their partners; and 4) adequate surveillance, monitoring and evaluation [4]. In 2008, ANC syphilis testing coverage, prevalence, treatment coverage, and congenital syphilis (CS) rate were added as indicators for country monitoring and reporting to the UNAIDS Global AIDS Monitoring (GAM) system [5] and are publicly reported in the WHO Global Health Observatory (GHO) [6]. This was followed by the WHO launch of the *Global Guidance on Criteria and Processes for Validation of Elimination of Mother-to-Child Transmission of HIV and Syphilis* in 2014. The targets countries need to achieve for elimination of MTCT of syphilis are 1) at least 95% of pregnant women attend antenatal care (ANC); 2) at least 95% of pregnant women in ANC receive syphilis screening; and 3) at least 95% of syphilis seropositive pregnant women receive adequate treatment [7].

Countdown to 2030 (CD2030) is a partnership among academic institutions, UN agencies, governments and other civil society members that provides independent analyses that aim to accelerate the achievement of the Sustainable Development Goals for ending preventable maternal, newborn and child deaths [8]. Its efforts are focused on 81 priority countries that account for more than 95% of maternal, and 90% of under-five, child deaths in the world. Moreover, CD2030 aims to utilize regional networks to build the capacity of countries to use evidence-based interventions to shape national plans and policies [9]. In 2018, CD2030 selected prevention of MTCT of syphilis as one of their initiatives and added the WHO ANC syphilis testing and treatment indicators to those which each *Countdown* country should monitor.

We aimed to evaluate current maternal and congenital syphilis surveillance systems in the 81 CD2030 countries through examination of two country-reported syphilis service coverage indicators: 1) percentage of pregnant women tested for syphilis and 2) percentage of seropositive pregnant women who were treated for syphilis and coverage of attendance of at least 4 antenatal care visits (ANC4). The aims of this analysis are to: describe coverage of these services in CD2030 countries towards the 95% targets required for WHO validation of CS elimination; highlight gaps in antenatal surveillance that may reflect service gaps and estimate the number of CS-associated stillbirths and other adverse birth outcomes due to service gaps within ANC and among women not attending ANC in CD2030 countries. Country use of the GAM reporting system for reporting these indicators is also described. Given the burden of maternal and neonatal disease in these countries, progress achieved in preventing CS in CD2030 countries will help to drive progress towards achieving the goals of the Global Strategy for Women, Children, and Adolescent Health [10] and the Sustainable Development Goals [8] related to improvements in maternal and newborn health.

## METHODS

We utilized the Global AIDS Monitoring (GAM) surveillance system, which collates country-reported HIV/AIDS outcome and coverage indicators. Since 2008, GAM has included four key syphilis indicators: (i) maternal syphilis screening coverage and (ii) maternal syphilis treatment coverage during ANC, (iii) syphilis seroprevalence among ANC attendees, and (iv) national reported CS case rates, data which are publicly available in GHO [6]. We focused our analysis on the 81 countries prioritized by CD2030. The most recent data reported by countries during the 2-year interval from 2016 and 2017 were utilized to describe the percentage of pregnant women who were screened and treated for syphilis by country over this time period.

We applied a CS prevention cascade to estimate the number of CS cases that could be attributed to a service gap at each level of ANC service (ie, ANC attendance, syphilis testing, and treatment) similar to methods used to estimate the global burden of CS [1]. This estimate utilized those CD2030 countries with numerator and denominator data for ANC4, syphilis screening, diagnosis and treatment for data years 2016 or 2017. United Nations estimates of live births were combined with a global estimate of stillbirth to generate an estimate of total number of pregnancies for each country [11-14]. ANC was defined as attendance at a minimum of four ANC visits (ANC4) with data obtained from the WHO Department of Reproductive Health and Research ANC4+ Global Database March 2019 [15].This database contains data that are extracted from publicly available sources. Aggregate data reported from 2016-2017 among included countries were utilized to estimate the sum of women in ANC4 who were tested and not tested, the seroprevalence of syphilis in women attending ANC4, and the sum of ANC4 women who were seropositive that were treated and not treated. The number of women who were in ANC4 and not tested and the number of women not in ANC4 were multiplied by the reported maternal syphilis seroprevalence from GAM and added to the women that tested positive in ANC4 but were not treated to estimate the number of WHO-defined congenital syphilis cases attributable to a missed opportunity in ANC4 [13,16]. To estimate burden of active, transmissible syphilis, a standard syphilis test type correction factor was applied to adjust for syphilis test positivity that could be due to previously treated syphilis consistent with global CS estimation methods [1,17]. Lastly, we applied the previously derived estimate that untreated cases of maternal syphilis among women in ANC4 and not in ANC4, incurred a 52% risk of adverse birth outcomes (ABOs) and a 21% risk of stillbirth, to estimate the total number of ABOs and stillbirths that were due to CS and could have been avoided with maternal syphilis screening and treatment in 2016-2017 amongst these CD2030 countries [1,13,16].

We used the WHO case definition of congenital syphilis pertaining to infants born to pregnant women with untreated syphilis as follows [7]:

*“*The WHO global surveillance case definition for congenital syphilis includes *A live birth or fetal death at >20 weeks of gestation or >500 g (including stillbirth) born to a woman with positive syphilis serology and without adequate syphilis treatment.*”

We assumed reported treatment coverage reflected the WHO definition of adequate treatment to prevent congenital syphilis defined as at least one injection of 2.4 million units of benzathine penicillin given at least 30 days prior to delivery. We also evaluated the reported syphilis diagnostic test types used, with a focus on the uptake of rapid syphilis testing over the two-year period 2016-2017. For evaluation of indicators and diagnostic test type, those countries not reporting any data for 2016 to 2017, or only reporting data prior to 2016, were considered “Missing/Unknown.” Microsoft Excel (Microsoft Inc, Seattle, WA, USA) was used for all calculations with results confirmed in SPSS V. 21 (IBM Inc, Armonk, NY, USA).

## RESULTS

Of 81 CD2030 countries, 53 (65%) reported one or more values for CS indicators into the GAM system during 2016-2017. Data on maternal syphilis testing coverage was provided by 52 (64%), screening positivity was reported by 49 (60%), treatment coverage was reported by 41 (51%) and syphilis test type was reported by 53 (65%) CD2030 countries. CS case rates were only reported by 13 (16%) CD2030 countries (**Table 1**). Of the 81 CD2030 countries, 40 (49%) reported data for all three indicators related to coverage of services (syphilis testing, test positivity, and treatment of seropositive women) during 2016 and 2017. Reported screening and treatment coverage is summarized in **Table 2**.

**Table 1:** Syphilis screening, positivity, and treatment coverage for 81 Countdown countries 2016-2017*

Country	Syphilis test type	WHO region	% ANC screening	Year % screening	% positive	Year % positivity	% treat	Year % treat	CS rate**	Year CS rate
Afghanistan	non-treponemal (RPR,VDRL)/treponemal (rapid tests, TPPA)	Eastern Mediterranean	14.3	2017	0.3	2017	100.0	2017		
Algeria	not reported	Africa								–
Angola	not reported	Africa								
Azerbaijan	not reported	Europe						–		
Bangladesh	patients positive on both: non-treponemal/treponemal	South East Asia	72.3	2017	0.0	2017	100.0	2017		–
Benin	non-treponemal (RPR,VDRL)|treponemal (rapid tests, TPPA)	Africa	3.1	2017	0.4	2017	100.0	2017		–
Bhutan	not reported	South East Asia						–		
Bolivia (Plurinational State of)	patients positive on both: non-treponemal/treponemal	Americas	96.0	2017	0.9	2017	100.0	2017		
Botswana	non-treponemal (RPR,VDRL)	Africa						–		–
Burkina Faso	non-treponemal (RPR,VDRL)|treponemal (rapid tests, TPPA)	Africa	100.0	2016	0.7	2016	100.0	2017	669	2017
Burundi	not reported	Africa						–		–
Cambodia	treponemal (rapid tests, TPPA)	Western Pacific	62.9	2017	0.0	2017	83.9	2017		–
Cameroon	Not reported	Africa								–
Central African Republic	non-treponemal (RPR,VDRL)|patients positive on both	Africa	56.1	2017	4.7	2017	97.4	2017		–
Chad	Not reported	Africa								–
Comoros	Not reported	Africa						–		–
Congo	non-treponemal (RPR,VDRL)	Africa	10.7	2016	0.6	2016		–		–
Côte d'Ivoire	treponemal (rapid tests, TPPA)	Africa						–		–
Democratic People’s Republic of Korea	not reported	South East Asia						–		–
Democratic Republic of the Congo	non-treponemal (RPR,VDRL)|treponemal (rapid tests, TPPA)|patients positive on both	Africa								–
Djibouti	not reported	Eastern Mediterranean						–		–
Dominican Republic	non-treponemal (RPR,VDRL)	Americas	42.2	2017	1.6	2017	54.1	2017		
Equatorial Guinea	not reported	Africa								
Eritrea	treponemal (rapid tests, TPPA)	Africa	97.2	2017	1.1	2017	100.0	2017		–
Ethiopia	not reported	Africa	44.6	2017	1.1	2017	100.0	2017		–
Gabon	non-treponemal (RPR,VDRL)|treponemal (rapid tests, TPPA)	Africa	31.1	2017	1.8	2017	100.0	2017		
Gambia	not reported	Africa				–				–
Ghana	non-treponemal (RPR,VDRL)|treponemal (rapid tests, TPPA)|patients positive on both	Africa	44.6	2017	3.0	2017	91.0	2017		–
Guatemala	non-treponemal (RPR,VDRL)|treponemal (rapid tests, TPPA)|patients positive on both	Americas	37.1	2017	0.1	2017	47.6	2017	9.2	2017
Guinea	treponemal (rapid tests, TPPA)	Africa	4.8	2017	5.4	2017	100.0	2017		–
Guinea-Bissau	not reported	Africa						–		–
Guyana	not reported	Americas								
Haiti	treponemal (rapid tests, TPPA)	Americas	92.5	2016	2.8	2016	89.8	2017		–
Honduras	non-treponemal (RPR,VDRL)|treponemal (rapid tests, TPPA)|patients positive on both	Americas	69.0	2017	0.2	2017			89.5	2017
India	non-treponemal (RPR,VDRL)	South East Asia	19.8	2017	0.1	2017	47.6	2017		–
Indonesia	patients positive on both: non-treponemal/treponemal	South East Asia	1.7	2017	3.2	2017	30.1	2016	1.2	2016
Iraq	not reported	Eastern Mediterranean						–		–
Jamaica	treponemal (rapid tests, TPPA)	Americas	90.0	2016	1.5	2016	70.9	2016	22.8	2016
Kenya	treponemal (rapid tests, TPPA)	Africa	85.7	2017	1.4	2017		–		–
Kyrgyzstan	patients positive on both: non-treponemal/treponemal	Europe	89.4	2017	0.0	2017	100.0	2017	3.2	2017
Lao People's Democratic Republic	not reported	Western Pacific		–	0.8	2009		–		–
Lesotho	non-treponemal (RPR,VDRL)	Africa	91.2	2017	2.4	2016				–
Liberia	treponemal (rapid tests, TPPA)	Africa								–
Madagascar	treponemal (rapid tests, TPPA)	Africa	28.8	2017	3.0	2017	61.0	2017		–
Malawi	treponemal (rapid tests, TPPA)	Africa	82.0	2017	1.0	2017	100.0	2016		–
Mali	non-treponemal (RPR,VDRL)	Africa	21.4	2017	6.1	2017	100.0	2016		–
Mauritania	not reported	Africa								–
Morocco	treponemal (rapid tests, TPPA)	Eastern Mediterranean	43.0	2017	1.3	2017	52.6	2017	22.5	2017
Mozambique	treponemal (rapid tests, TPPA)	Africa	71.9	2017	4.6	2017				
Myanmar	patients positive on both: non-treponemal/treponemal	South East Asia	31.2	2017	0.2	2017	71.4	2017		–
Namibia	non-treponemal (RPR,VDRL)	Africa	97.9	2017	2.1	2017			6.5	2017
Nepal	non-treponemal (RPR,VDRL)|treponemal (rapid tests, TPPA)	South East Asia	0.3	2016		2016	16.7	2016	0	2017
Nicaragua	non-treponemal (RPR,VDRL)|treponemal (rapid tests, TPPA)	Americas	76.2	2017	0.1	2017	98.3	2017	3	2017
Niger	non-treponemal (RPR,VDRL)|treponemal (rapid tests, TPPA)	Africa	19.7	2016	0.9	2016	100.0	2016		–
Nigeria	non-treponemal (RPR,VDRL)	Africa	16.1	2017	0.8	2017	74.8	2017		–
Pakistan	not reported	South East Asia								
Panama	non-treponemal (RPR,VDRL)|treponemal (rapid tests, TPPA)|patients positive on both	Americas	92.7	2017	1.8	2017	83.8	2017	530	2017
Papua New Guinea	treponemal (rapid tests, TPPA)	Western Pacific	45.6	2017	6.8	2017	76.9	2017		–
Paraguay	non-treponemal (RPR,VDRL)|treponemal (rapid tests, TPPA)|patients positive on both	Americas	92.7	2017	1.9	2017	66.8	2017	287.6	2017
Philippines	patients positive on both: non-treponemal/treponemal	Western Pacific	11.7	2017	0.9	2017	43.3	2017		–
Rwanda	not reported	Africa						–		–
Senegal	patients positive on both: non-treponemal/treponemal	Africa	39.0	2017	2.4	2017	62.4	2017		–
Sierra Leone	not reported	Africa	8.3	2017	0.1	2017	100.0	2017		–
Solomon Islands	non-treponemal (RPR,VDRL)	Western Pacific					100.0	2017		–
Somalia	treponemal (rapid tests, TPPA)	Eastern Mediterranean	43.4	2016	1.3	2016				–
South Africa	treponemal (rapid tests, TPPA)	Africa	100.0	2017				–		–
South Sudan	non-treponemal (RPR,VDRL)	Africa	100.0	2017	7.6	2017		–		–
Sudan	not reported	Eastern Mediterranean						–		–
Suriname	not reported	Americas		–				–		
Swaziland	not reported	Africa	85.2	2016	2.3	2016		2016		–
Tajikistan	patients positive on both: non-treponemal/treponemal	Europe	100.0	2016	0.0	2016	100.0	2016	3.5	2017
Timor-Leste	not reported	South East Asia						–		–
Togo	treponemal (rapid tests, TPPA)	Africa	9.3	2017	2.2	2017	100.0	2017		–
Turkmenistan	not reported	Europe								
Uganda	non-treponemal (RPR,VDRL)	Africa	43.3	2016	2.9	2016		–		–
United Republic of Tanzania	non-treponemal (RPR,VDRL)	Africa	42.0	2017	1.8	2017	56.9	2016		–
Uzbekistan	not reported	Europe						–		–
Venezuela (Bolivarian Republic of)	non-treponemal (RPR,VDRL)	Americas	30.6	2016	2.0	2016				
Yemen	not reported	Eastern Mediterranean		–				–		–
Zambia	non-treponemal (RPR,VDRL)/Treponemal (rapid tests, TPPA)	Africa	56.0	2017	3.5	2016	100.0	2017		–
Zimbabwe	treponemal (rapid tests, TPPA)	Africa	98.7	2017	1.9	2017	78.4	2017		–

ANC – antenatal care, WHO – World Health Organization, CS – Congenital syphilis, RPR – Rapid-Plasma-Reagin, VDRL – Venereal Disease Research Laboratory, TPPA – *Treponema pallidum* particle agglutination assay

*Country-reported data into the UNAIDS Global AIDS Monitoring System (GAM) 2016-2017 [6].

†CS rate reported as CS cases per 100 000 live births.

**Table 2:** Reported performance coverage of antenatal care (ANC) syphilis screening and treatment among Countdown countries (N = 81)

ANC Syphilis Service Indicator Coverage	95% coverage*, n (%)	75%-94% coverage, n (%)	50%-74% coverage, n (%)	<50% coverage, n (%)	Data not reported
Syphilis screening	9 (11)	10 (12)	6 (7)	27 (33)	28 (35)
Syphilis treatment†	21 (26)	8 (10)	8 (10)	4 (5)	40 (49)

*WHO target for validation of ending mother-to-child transmission.

†Treatment coverage among those found to be syphilis test positive

Importantly, many countries reporting high treatment coverage reported very low syphilis screening coverage in ANC. Of 28 countries reporting ≥75% treatment coverage, 13 (43%) had <50% coverage for testing and an additional four (14%) had moderate (50%-74%) testing coverage. ANC4 coverage notwithstanding, four CD2030 countries (Bolivia, Burkina Faso, Eritrea, and Tajikistan) reported met the WHO targets of 95% coverage for both syphilis testing and treatment.

**Figure 1:**
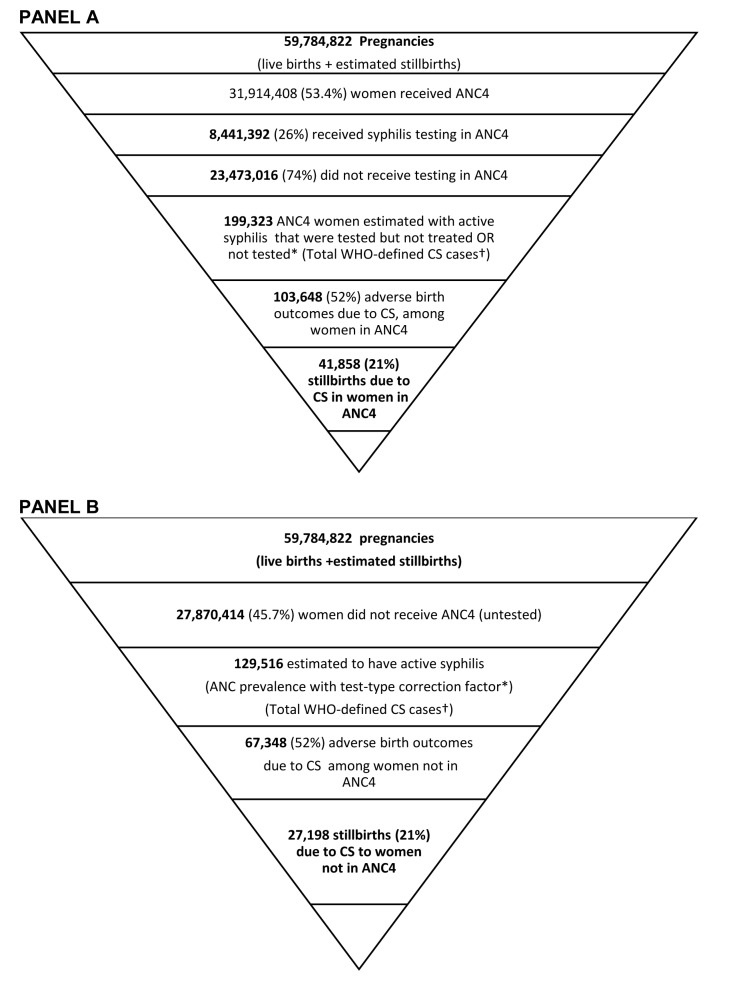
Estimated congenital syphilis cases associated with missed opportunities for testing and treatment. **Panel A.** Receipt of Antenatal Care, at least four visits (ANC4). *Test type correction factor applied to percent testing positive and ANC women untested but assumed positive based on prevalence of tested proportion. **Panel B.** No receipt of ANC. *The same test-type correction factor applied for this proportion of women not attending ANC4 as was applied for those in ANC4. Country-reported maternal syphilis prevalence of women in ANC applied to this calculation (GAM). †The WHO global surveillance case definition for congenital syphilis is *A live birth or fetal death at >20 weeks of gestation or >500 g (including stillbirth) born to a woman with positive syphilis serology and without adequate syphilis treatment*” [6].

Analysis of the 40 CD2030 countries with data for ANC4, syphilis testing, seroprevalence and treatment was performed to build a CS prevention cascade and estimate the number of CS cases that were attributable to each service delivery gap (N = 59 784 822 pregnant women) (**Figure 1**, Table S1 in the **Online Supplementary Document**). Of 31 914 408 (53%) pregnant women receiving at least four ANC visits in these 40 countries 8 441 392 (26%) were tested for syphilis, while 23 473 016 (74%) pregnant women were in ANC4 but not tested. Based on reported maternal syphilis seroprevalence and testing and treatment coverage, an estimated 199 323 women attended ANC4 with active syphilis but were either not tested, or tested but not treated (WHO-defined CS cases). As previously estimated, 52% of untreated cases of maternal syphilis are estimated to result in ABOs including stillbirths [13,16]. Applying these estimates, the ANC4 service gaps above resulted in a total of 103 648 ABOs with 41 858 of these occurring as stillbirths among women attending ANC4 in the 40 *Countdown* countries with complete data for years 2016 or 2017. Women in the 40 countries that did not attend ANC4 (n = 27 870 414) contributed to the CS burden with an additional 129 516 WHO-defined CS cases with 67 348 estimated ABOs of which 27 198 of were stillbirths (**Figure 1**). In total, 170 996 ABOs were estimated with 69 056 occurring as stillbirths in these 40 countries. The remaining 41 *Countdown* countries (81-40 = 41) did not have data available for this analysis.

During 2016 and 2017, 53 (65%) *Countdown* countries reported the type(s) of syphilis diagnostic tests used for the two-year period in ANC clinics. Of these, 17 (32%) reported use of rapid treponemal-based syphilis tests; 13 (24.5%) reported use of non-treponemal diagnostic tests alone such as rapid plasmin reagin (RPR) or venereal disease research laboratory (VDRL) tests, and 23 (43%) reported use of non-treponemal diagnostic tests followed by confirmatory treponemal testing (using *Treponema pallidum* particle agglutination (TPPA) or *Treponema pallidum* haemagglutination (TPHA) assay (**Table 1**).

## DISCUSSION

Only half of these 81 *Countdown* countries entered data on syphilis testing and treatment coverage into the UNAIDS GAM system in 2016 or 2017. For many countries, reported ANC syphilis services require scale-up to achieve elimination of maternal-to-child-transmission (EMTCT) by 2030. Using a pre-existing surveillance system, we were able to identify 4 countries that have reported reaching the WHO syphilis service coverage targets for validation of elimination of vertical transmission of syphilis for both testing and treatment of maternal syphilis in 2016-2017. Using a cascade-type analysis, we identified opportunities to improve syphilis testing and treatment coverage that would prevent a large number of preventable ABOs and stillbirths. These analyses demonstrate opportunities for antenatal syphilis surveillance and service improvements as part of congenital syphilis elimination efforts.

By utilizing the UNAIDS GAM system, our data can serve as an evaluation of the current state of coverage of antenatal syphilis surveillance indicators as reported by these 81 priority CD2030 countries. These data allow CD2030 to specifically tailor their congenital syphilis prevention and elimination efforts. Rather than creating a new surveillance system, efforts can be centered on improving reporting and analysis of data reported to UNAIDS GAM, and evaluating reporting and coverage trends over time, as well as surveillance gaps. For this to happen, more CD2030 countries could be supported to report ANC syphilis indicator data to GAM, and those that are now reporting could do so more completely and consistently [5].

It is important to note that the collective targets for ANC coverage, antenatal syphilis testing and antenatal syphilis treatment must be met to achieve the goal of elimination of CS, and women must first have access to, and attend, ANC in order to take advantage of these preventive services. For this reason, reported coverage of each indicator must be interpreted in the context of the other indicators. Nearly, one-third of CD2030 countries (25, 30.9%) reported >75% maternal syphilis treatment coverage, however, many of these countries had very low testing coverage. These countries may be doing a good job at treating pregnant women who are seropositive for syphilis, but they are not doing a good job at screening this population, and thus likely have not seen improvements in CS rates.

As the targets for elimination of CS are predicated on women accessing ANC, the cascade analysis of missed opportunities for prevention demonstrates how service gaps at each level contribute to the total missed opportunities for intervention and CS rate reduction. Using the GAM data for 2016-2017, we were able to estimate the ABOs and stillbirths that are due to missed opportunities within and outside of ANC, data which can be used to support surveillance and service improvements. Moreover, it is important to note that this estimate likely substantially underestimates the number of ABOs and stillbirths attributable to limited syphilis testing and treatment coverage in the CD2030 countries as our cascade analysis was limited to only 40 of 81 CD2030 countries with complete data for each indicator. It is plausible that even with high coverage of syphilis testing, treatment rates may be lower, even in countries that have prioritized efforts to eliminate congenital syphilis, due to shortages of benzathine penicillin during this same time period. Of note, 29 out of 41 countries with shortages in benzathine penicillin between 2014 and 2016 were also CD2030 countries which may explain lower treatment coverage for some [18]. Lastly, it is possible that by virtue of collecting and reporting on these data, these CD2030 countries may in fact, represent the highest coverage for these indicators, which would mean our results grossly underestimate the reality that exists in the remaining CD2030 countries.

The methods used for estimation of CS cases, ABOs and stillbirths in this analysis were similar to those used for global estimation of CS [1]. This analysis differs from that of the recent global CS estimation in that the data input for ANC coverage here was ANC4 whereas ANC1 (at least one ANC visit) was used for the 2016 global CS estimates. The use of ANC4 reflects the WHO modification of the minimum number of ANC visits to 8 contacts in order to achieve effective care during pregnancy, which is now the ANC coverage indicator monitored for CD2030 countries [19]. Data sources for live births were different. Modelling methods used for global estimates of maternal syphilis prevalence were not used here. We assumed reported treatment was adequate for CS prevention and did not estimate residual CS cases occurring due to inadequate or late treatment. Thus, the recently reported 2016 WHO estimates of CS that include these countries cannot be compared to estimates presented here. We readily acknowledge that some countries may collect ANC syphilis screening and treatment coverage but not report into the GAM system. Considering the countries that do utilize GAM, further analyses are needed to definitively identify and address the underlying circumstances to explain high treatment coverage rates in the face of low coverage of testing. For a myriad of reasons, many women do not access ANC. Within ANC, syphilis screening may not be offered due to limited or no testing capacity or it may be offered but at an additional cost. Stock-outs of syphilis test kits and reagents are common, and the need to present to an off-site laboratory for testing may be an additional hurdle. Women may be lost to follow up for treatment after diagnosis of syphilis is made for several reasons. This may include cases where women need to return to a laboratory to obtain results, require outside treatment referrals, have additional costs to receive treatment. Countries may face penicillin shortages resulting in no treatment or providers may use alternative regimens which are not recommended or lack effectiveness data for prevention of CS [18]. Lastly, even those women who are screened and appropriately treated for syphilis remain at risk for re-infection if their partners are not appropriately treated.

Through evaluation of diagnostic test type, it is possible to highlight the use and benefits of rapid syphilis testing in low- and middle-income countries. Among countries reporting test type, approximately one-third (32%) use rapid syphilis tests during ANC. Rapid syphilis testing has the advantage of providing same day results with the opportunity to treat at the time of diagnosis. Utilization of rapid syphilis testing could allow countries to both increase the number of pregnant females screened, while also increasing their treatment coverage within ANC settings where service coverage can be evaluated and monitored. Two rapid dual HIV/syphilis test kits have now received WHO pre-qualification [20] Use of these test kits can result in immediate scale up of syphilis screening alongside that of HIV with the option of same visit treatment for women testing positive for syphilis [21].

These data and this analysis have limitations. Data reporting into the GAM system were inconsistent over the years, some data were implausible, and there are numerous CD2030 countries that did not provide any data into this system. Additional data sources for maternal syphilis screening and treatment of pregnant women may be available but not included in the UNAIDS GAM reporting. It is difficult to know if only the surveillance system is lacking in these countries, or if the coverage of these indicators is also lacking. Same day testing and treatment through the use of point-of-care rapid syphilis tests among women attending only one ANC visit is possible and thus our results could be overestimates for countries where this service is in place. Our analysis of these data are purely descriptive in nature.

These 81 CD2030 countries account for more than 95% of maternal and 90% of under-five child mortality in the world [22]. As a result, uptake of effective syphilis interventions in pregnancy by these countries is well placed to decrease overall levels of preventable, adverse maternal and neonatal outcomes, and eliminate congenital syphilis. To our knowledge this study represents the first evaluation of antenatal syphilis screening and treatment coverage in the 81 CD2030 countries using data reported into the UNAIDS GAM system, as well as the first estimate of ABOs and stillbirths attributable to these service gaps in CD2030 countries where data are available. We encourage countries to improve ANC syphilis screening and treatment coverage and to prioritize the use of the UNAIDS GAM system to capture and monitor progress towards EMTCT [7]. Information on the downstream effects of missed opportunities to screen and treat women for syphilis during ANC provide a rich source of evidence for scale up [23,24].

## Additional material

Online Supplementary Document
